# First case of systemic phaeohyphomycosis due to *Cladophialophora bantiana* in Slovakia

**DOI:** 10.1099/jmmcr.0.002659

**Published:** 2014-12-01

**Authors:** Martina Sládeková, Miroslava Pőczová, Miroslav Gašpar, Ivan Vojtech, Jaroslava Chupáčová, Helena Bujdáková, Katarína Šoltýs, Tomáš Szemes, Juraj Hanzen

**Affiliations:** ^1^​ HPL Ltd., Department of Mycology, Bratislava, Slovak Republic; ^2^​ University Hospital, Clinic of Infectology and Geographic Medicine, Bratislava, Slovak Republic; ^3^​ Comenius University in Bratislava, Faculty of Natural Sciences, Department of Microbiology and Virology, Slovak Republic; ^4^​ Comenius University in Bratislava, Faculty of Natural Sciences, Department of Molecular Biology, Slovak Republic; ^5^​ Geneton Ltd., Bratislava, Slovak Republic

**Keywords:** *Cladophialophora bantiana*, brain abscess, disseminated infection

## Abstract

**Introduction:**

Melanized or dematiaceous fungi are associated with a wide variety of infectious syndromes. *Cladophialophora bantiana* is one of the most common and dangerous neurotropic fungi, able to cause brain abscess and disseminated infection.

**Case Presentation:**

We report a new case of phaeohyphomycosis brain abscesses caused by *C. bantiana* in Slovakia. The patient was a 63-year-old man having undergone heart transplantation, with dyspnoea, left-sided bronchopneumonia and fevers. CT (computed tomography) and MRI (magnetic resonance imaging) of the brain revealed numerous abscesses. Bacterial infection was proven by neither the growth of bacteria in culture nor the presence of bacterial antigens. Direct microscopy of the pus from the brain abscess showed Gram-positive hyphae. The isolate was finally identified as *C. bantiana* based on morphological and physiological features, and on DNA sequence analysis.

**Conclusion:**

In spite of appropriate therapy, neurological complications and accelerated respiratory insufficiency resulted in the patient’s death. Concerning clinical manifestation of the brain phaeohyphomycosis that can sometimes be a problem to distinguish from malignancy, physicians should also assume infection caused by this serious pathogen.

## Introduction

Phaeohyphomycosis refers to infections caused by darkly pigmented fungi. These fungi contain melanin pigments in their cell walls and spores. Dematiaceous fungi are mainly saprophytic, but at present, over 150 species and about 70 genera of dematiaceous fungi are known to cause various diseases and the number of potential pathogens has increased (Ajantha & Kulkarni, 2011). The spectrum of phaeohyphomycosis symptoms ranges from solitary subcutaneous nodules associated with local trauma, to mycetoma, to life-threatening infections, such as brain abscesses and disseminated disease (George *et al.*, 2008). *C*. *bantiana* is one of the most common pathogens that cause cerebral phaeohyphomycosis (Levin *et al.*, 2004). To date, more than 70 cases of *C*. *bantiana*-induced infections have been reported (Li & de Hoog, 2009; Aher & Rastogi, 2012). We report the first case of disseminated disease caused by *C*. *bantiana* in Slovakia.

## Case report

A 63-year-old man having received a heart transplant due to dilated cardiomyopathy (May 2012) was hospitalized at the Department of Internal Medicine in Ilava hospital, Slovakia (December 2012). The patient had dyspnoea, left-sided bronchopneumonia and fevers. He was treated with amoxicillin/clavulanate and ciprofloxacin. The left-sided hemiparesis becoming hemiplegia evolved during the hospitalization. The CT (computed tomography) of the brain revealed numerous focal changes in the brain parenchyma, and suspected infra- and supratentorial metastatic lesions.

**Fig. 1.**
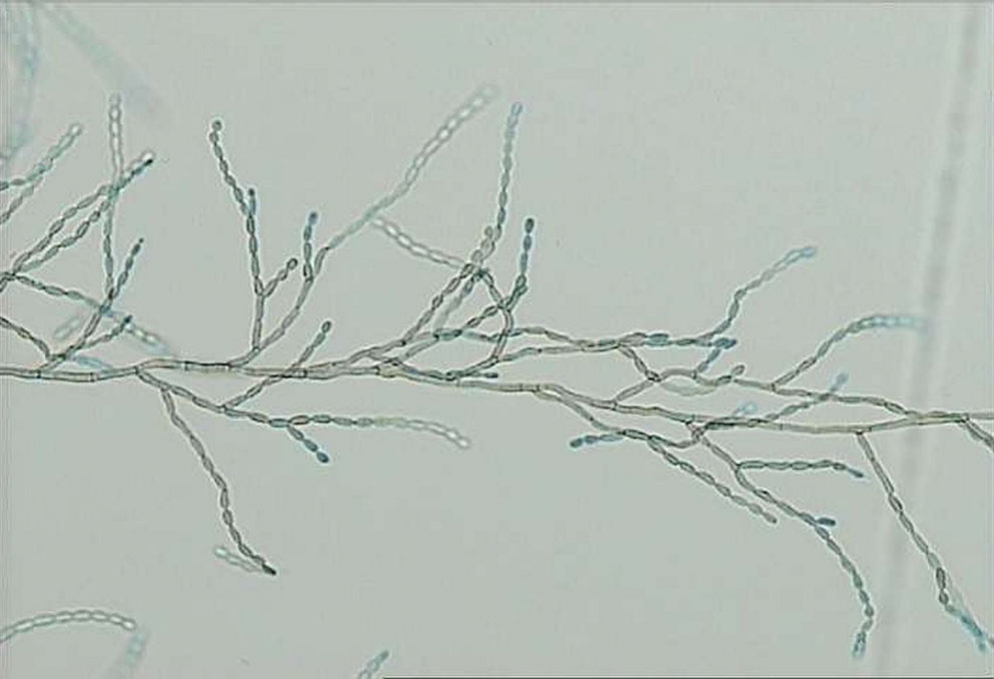
Slide culture in lactophenol preparation showing dark-walled, septate hyphae and long chains of single-celled conidia (original magnification ×200).

The patient was transferred to the ICU (Intensive Care Unit) of the Clinic of Infectology and Geographic Medicine (January 2013). His pulse was 75 BPM and blood pressure was 125/70 mm mercury. The patient had a headache, cough and he was afebrile, with hemiparesis on the left lower limb. Anti-oedemotous and switched antibiotic therapy by third-generation cephalosporin and metronidazole was initiated. The investigations were complemented by MRI (magnetic resonance imaging) of the brain, which did not prove metastases and showed numerous abscesses with a strong oedema supra- and infratentorially in the posterior cranial fossa. In the quest for the brain abscess etiology, biological materials were collected for serological and microbiological tests. Blood culture was sterile. The cerebrospinal fluid was negative for any bacteria and bacterial antigens (*Neisseria meningitidis* serotypes a, b, c, y, w135; *Streptococcus* group B; *Haemophilus influenzae* serotype b; *Streptococcus pneumoniae*; *Escherichia coli* K1). The mycological serology, antigens of *Cryptococcus neoformans*, *Aspergillus* spp. and *Candida* spp., was negative as well. The pus from the skin lesion was examined by the Gram staining technique, showing the presence of Gram-positive hyphae. Native preparation and Lactophenol Cotton Blue staining of the material from the skin biopsy revealed the same observation – numerous darkly pigmented fungal elements with septa. As a bacteriological examination was negative, the samples were further cultured on Sabouraud dextrose agar (SDA) (HPL SERVIS Ltd. Nesvady, Slovak Republic) or Sabouraud dextrose broth (SDB) (HPL SERVIS Ltd. Nesvady, Slovak Republic) with antibiotics. The SDA and SDB were incubated at both 25 °C and 37 °C. The growth rate was moderate and it took about 4–5 days of incubation. After that time, velvety, olivaceous, grey colonies with olivaceous, black reverse could be observed (Figs S1 and S2). Colonies did not produce any diffusible pigment. The slide culture of the mould was incubated at 25 °C and after 5–7 days showed dark-walled, septate hyphae with poor branching. The one-celled oval conidia (7.5–11 µm×2.5–4 µm) were smooth-walled and pale brown without pigmented hila. They formed long, coherent, sessile, lateral or terminal chains on undifferentiated hyphae (Fig. 1). The fungus grew on SDA and malt agar at 42 °C, with the optimal growth temperature between 35 and 37 °C. The mould was urease positive, growth on 10% NaCl agar was negative and colonies could grow on media containing cycloheximide. Based on these results, the isolate was identified in our laboratory as *C. bantiana*. To confirm this observation, we performed DNA sequencing-based identification. DNA was extracted using a Qiagen kit according to the manufacturer’s protocol and used for PCR with a specific set of primers for the internal transcribed spacer (ITS) regions of the fungal rRNA gene ITS1 (5′-TCCGTAGGTGAACCTGCG-3′) and ITS4 (5′-TCCTCCGCTTATTGATATGC-3′) (Invitrogen) according to Fujita *et al*. (2001). The PCR products were sequenced twice in both directions and the consensus sequence (596 nt) was used for subsequent blastn analysis (Zhang *et al*., 2000) against the nucleotide database with standard blast settings. The consensus obtained was aligned with the sequence of the ITS1 and ITS2 region of *C. bantiana* strain 1394 (sequence ID GQ258793), which had the highest score (1037 bits). Over 578 nt of the alignment, 99% identity, 2 gaps and 8 mismatching nucleotides were observed. Other *C. bantiana* strain ITS sequences represented all 13 highest-scoring blast matches. Finally, the isolate was identified as *C. bantiana* based on morphological and physiological features, and on the DNA sequence analysis. The sequence obtained was registered in the genetic sequence database GenBank under accession number KM525668.

**Fig. 2.**
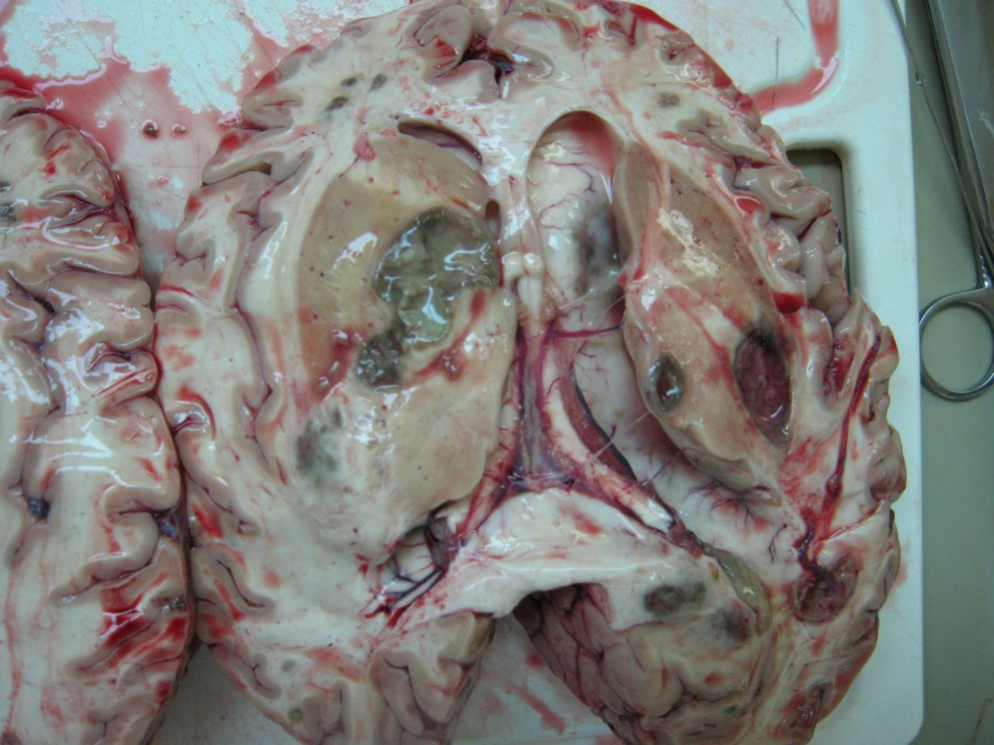
Olivaceous, brown lesions in brain autopsy material.

Susceptibility testing to selected antimycotics was performed on RPMI medium by quantitative assay for determining the MIC (MIC Test Strip; Liofilchem). After 48–72 h of cultivation at 30 °C, the MICs of 0.006 mg posaconazole l^−1^, 0.125 mg voriconazole l^−1^, 0.016 mg itraconazole l^−1^ and 0.75 mg amphotericin B l^−1^, were evaluated. There are no standard guidelines for antifungal therapy and until now, it has not been confirmed that therapy alone can improve survival (Ajantha & Kulkarni, 2011). The current recommendation for eradication of disease associated with dematiaceous fungi is a combination of total surgical excision followed by systemic antifungal therapy (Huang *et al.*, 2011; Aher & Rastogi, 2012). Our patient received an intravenous administration of amphotericin B and intense anti-oedematous therapy. The status of the patient was not improved, retrograded neurological complications and accelerated respiratory insufficiency resulted in the patient’s death one month later (February 2013). As the death of the patient was classified as being due to systemic fungal infection, a pathological-anatomic autopsy was recommended. Finally, *C. bantiana* was isolated from autopsy material taken from the lung, brain (Fig. 2) and skin.

## Discussion

Fungal infections of the CNS (central nervous system) and disseminated fungal infections are often associated with severe immunodeficiency, but primary cerebral phaeohyphomycosis appears to be an exception to this rule (Revankar *et al.*, 2004). Additionally, some cases occur in immunocompetent individuals as well (Banerjee *et al*., 2002). Besides immunocompromised patients with underlying malignancy, patients with organ transplantations and acquired immunodeficiency syndrome are further candidates for acquiring fungal infections. CNS infections by melanized fungi have increased in recent years and have often been caused by black mould e.g. *Exophiala dermatitidis*, *Ramichloridium mackenzie* and *Ochroconis gallopavum*, etc. (Revankar *et al*., 2004; Aher & Rastogi, 2012).

*C*. *bantiana* is also a melanized hyphomycetous fungus. Its natural habitats are soil and rotten plant material (e.g. decaying lumber, vegetation, sawdust, brick walls). It is distributed worldwide, although the exact ecological niche of the fungus is unknown (Huang *et al*., 2011). This organism generally prefers a warmer climate with high humidity (Ajantha & Kulkarni, 2011). The first samples of *C. bantiana* from an indoor environment originated from hot-tub water samples taken from a retirement community in the USA (Jurjevic *et al*., 2013). *C. bantiana* is most probably acquired via inhalation. Rarely occurring cutaneous or subcutaneous infections result from traumatic inoculation (Werlinger & Yen, 2005). *C*. *bantiana* is a highly neurotropic dematiaceous fungus that can cause human brain abscesses. One of the factors that may be responsible for pathogenicity is the presence of melanin. Several studies mentioned a high mortality, even in treated cases (Banerjee *et al*., 2002; Levin *et al*., 2004). The predisposing factors include organ transplantation, glucocorticoid treatment, diabetes mellitus, neutropenia, direct inoculation, ocular injury, intravenous drug abuse, etc. (Levin *et al*., 2004; Revankar *et al*., 2004; Aher & Rastogi, 2012).

In our case, the patient was addicted to alcohol even after the heart transplant due to dilated cardiomyopathy. He received the immunosuppressive drugs tacrolimus, mycophenolate and prednisone, and treatment with broad-spectrum antibiotics.

The site of fungal entry is not well understood for solid-organ transplant recipients with cerebral phaeohyphomycosis. The most probable routes include hematogenous dissemination from a pulmonary source, but local spread from the sinuses or ears could also account for brain abscesses (Revankar *et al.*, 2004; Ajantha & Kulkarni, 2011; Huang *et al.*, 2011). In the presented case, the patient had multiple brain abscesses in intra- and supratentorial localization documented by CT, accompanied by headache followed by focal neurological deficit. In scope of finding the etiology of the brain abcesses, a broad spectrum of serological, bacteriological and mycological examinations was performed. From the brain biopsy and from the skin lesions on the right side of the back we isolated and identified *C*. *bantiana*. Treatment of the infection was very problematic. Combined antifungal chemotherapy, surgical debridement and careful immunological interventions are strongly recommended for treating cerebral phaeohyphomycosis (Li & de Hoog, 2009). At the time when *C. bantiana* was identified, the infection was already disseminated. The patient fell into a coma despite intravenous administration of amphotericin B. Treatment failure for the patient could be associated with intensive immunosuppressive therapy, bronchopneumonia, and his overall bad health status. Additionally, fungal brain abscess carries a poor prognosis, regardless of the underlying disease (Revankar, 2011; Martínez–Lamas *et al*., 2013).

Clinical manifestation of the brain phaeohyphomycosis can be problematic to distinguish from malignancy, thus physicians do not always expect fungal infection. Therefore, a diagnosis could be delayed (about one month), which can be too late for total surgical excision of the abscess. Our study confirmed, in agreement with previous observations, that *C. bantiana* is able to cause life-threatening infections and should be assumed a serious pathogen (Huang *et al*., 2011).

## Ethics

No ethical principles and rules were violated in the work presented. There were no experiments on humans or animals. The patient mentioned in our case report stays in anonymity.

## Abbreviations

CT: computed tomography.
